# Conversion to lanthanum carbonate monotherapy effectively controls serum phosphorus with a reduced tablet burden: a multicenter open-label study

**DOI:** 10.1186/1471-2369-12-49

**Published:** 2011-09-30

**Authors:** Nirupama Vemuri, Michael F Michelis, Albert Matalon

**Affiliations:** 1 Department of Medicine (Nephrology), Sierra View District Hospital, Porterville, CA, USA; 2 Department of Medicine, Division of Nephrology, Lenox Hill Hospital, New York, NY, USA; 3 Department of Medicine (Nephrology), New York University School of Medicine, New York, NY, USA

## Abstract

**Abstract:**

**Background**

Lanthanum carbonate (FOSRENOL^®^) is an effective, well-tolerated phosphate binder. The ability of lanthanum to reduce serum phosphorus levels to ≤5.5 mg/dL in patients with end-stage renal disease (ESRD) was assessed in a clinical practice setting.

**Methods**

A 16-week, phase IV study enrolled 2763 patients at 223 US sites to evaluate the efficacy of lanthanum carbonate in controlling serum phosphorus in patients with ESRD, and patient and physician satisfaction with, and preference for, lanthanum carbonate after conversion from other phosphate-binder medications. Patients received lanthanum carbonate prescriptions from physicians. These prescriptions were filled at local pharmacies rather than obtaining medication at the clinical trial site. Changes from serum phosphorus baseline values were analyzed using paired *t *tests. Patient and physician preferences for lanthanum carbonate versus previous medications were assessed using binomial proportion tests. Satisfaction was analyzed using the McNemar test. Daily dose, tablet burden, and laboratory values including albumin-adjusted serum calcium, calcium × phosphorus product, and parathyroid hormone levels were secondary endpoints.

**Results**

Serum phosphorus control (≤5.5 mg/dL) was effectively maintained in patients converting to lanthanum carbonate monotherapy; 41.6% of patients had controlled serum phosphate levels at 16 weeks. Patients and physicians expressed markedly higher satisfaction with lanthanum carbonate, and preferred lanthanum carbonate over previous medication. There were significant reductions in daily dose and daily tablet burden after conversion to lanthanum carbonate.

**Conclusions**

Serum phosphorus levels were effectively maintained in patients converted from other phosphate-binder medications to lanthanum carbonate, with increased satisfaction and reduced tablet burden.

**Trial Registration:**

ClinicalTrials.gov: NCT0016012

## Background

Uncontrolled serum phosphorus is strongly associated with increased mortality in patients with end-stage renal disease (ESRD) [1]. Because of this, the Kidney Disease Outcomes Quality Initiative (KDOQI) proposed a target serum phosphorus range of 3.5 to 5.5 mg/dL for ESRD patients, which could be achieved by a combination of dialysis, dietary restrictions, and adherence to phosphate-binder medication regimens [2].

Many patients receiving maintenance dialysis do not achieve target serum phosphorus levels for a variety of reasons, including the complexity of their medical condition, poor adherence to dietary restrictions, and poor adherence to phosphate-binder therapy [3]. Reasons for nonadherence to medication regimens have been shown to be complex and multifactorial [4-7], but the high daily tablet burden associated with most phosphate-binder medications may be a contributing factor [8,9]. Dialysis patients may be required to take more than 15 different medications daily for various comorbid conditions. The complex dosing schedules that result may reduce adherence to dosing regimens [10]. Any means of simplifying treatment by reducing daily tablet burden is theoretically a positive step toward improving phosphate control in patients with ESRD.

Lanthanum carbonate is a non-calcium phosphate binder with proven efficacy in the treatment of hyperphosphatemia in patients with ESRD [11,12]. In vitro studies suggest that lanthanum carbonate has a stronger affinity for phosphate ions compared with other binders [13]. This potential for lower doses and fewer tablets may facilitate improved patient adherence, thereby contributing to reduced morbidity and mortality by improving serum phosphorus control rates.

This study assessed the efficacy of lanthanum carbonate in patients with ESRD requiring treatment for hyperphosphatemia in a clinical practice setting and patient and physician satisfaction with, and preference for, lanthanum carbonate compared with previous phosphate binders. Total daily dose and tablet burden of lanthanum carbonate compared with previous phosphate-binder therapies and the safety and tolerability of lanthanum carbonate treatment were also evaluated.

## Methods

### Patients

Adult patients (≥18 y) with ESRD requiring treatment for hyperphosphatemia were included in this study. Patients receiving any investigational agent within 30 days before screening were excluded, as were pregnant or lactating women. Patients could withdraw from the study at any time, but withdrawal was mandatory if they developed intercurrent illness or an adverse event that precluded further study participation, required an alternative phosphate binder, became pregnant, or if discontinuation was recommended by the investigator. This study was conducted in adherence with the ethical principles set forth in the Declaration of Helsinki (1989) and with local laws and regulations relevant to the use of new therapeutic agents. The protocol was approved for each center by an Institutional Review Board. All patients provided written, informed consent before participating in the study.

### Study Design

This was an open-label, phase IV, multicenter study conducted in the United States between January and December 2005. There was an initial screening visit and a 1-week observation period using existing phosphate-binder therapy during which no dose modifications were permitted. After the observation period, patients immediately began a 12-week lanthanum carbonate titration period without washout. At the end of the titration period, patients continued lanthanum carbonate treatment during a 4-week maintenance period. A follow-up interview (telephone or face-to-face) was conducted 30 days after the last dose of study medication to identify emergent serious adverse events.

### Study Medication

The original formulation of lanthanum carbonate was supplied by patients' local pharmacies using the TrialCard^® ^prescription program (TrialCard Inc, Cary, NC, USA); each card was study-specific and individually coded. A prescription was required for medication disbursement, and the physician was required to activate the card at the baseline visit (at the beginning of the titration period) and at visit 2 (beginning of the 4-wk maintenance period). All patients, regardless of prior therapy, received an initial daily dose of 1500 mg (250-mg or 500-mg tablets) in divided doses with meals that was adjusted, if necessary, in 2 to 3 weekly increments of 750-mg per day, up to the recommended maximum dose of 3750 mg per day to achieve serum phosphorus levels within the KDOQI guidelines target range of 3.5 to 5.5 mg/dL [1.13-1.78 mmol/L]).

### Assessments

The intent-to-treat (ITT) population included all patients who received at least 1 dose of study medication and underwent at least 1 primary efficacy evaluation. The primary objectives of the study were to evaluate efficacy and patient and physician satisfaction and preference. Predialysis serum phosphorus was measured at screening, at baseline visit, and at the end of the titration (week 12) and maintenance (week 16) periods of lanthanum carbonate treatment. A questionnaire that assessed satisfaction with phosphate-binder medication was completed by the patient and physician at baseline and at weeks 12 and 16 using a 7-item questionnaire for patients and a 6-item questionnaire for physicians; questions were rated on a 4-point Likert scale with answers ranging from "strongly agree" to "strongly disagree." For patients taking medication for hyperphosphatemia before participating in the study, a product preference questionnaire was completed by patients and physicians to determine preference for lanthanum carbonate or previous medication at weeks 12 and 16 using a 6-item questionnaire for patients and a 7-item question for physicians that addressed various aspects of treatment and overall preference.

Secondary endpoints included tablet burden and daily dose of phosphate-binder medication. These 2 parameters were assessed for all patients at screening, at baseline, weeks 12 and 16, and visits during which a new prescription indicating a different daily dose and tablet number was provided in accordance with titration procedures. Serum parathyroid hormone (PTH), corrected (ie, albumin-adjusted) serum calcium, and calcium × phosphorus (Ca × P) product were also measured at screening, baseline visit, and weeks 12 and 16 of treatment with lanthanum carbonate. These laboratory assessments were also measured bimonthly at visits during the 12-week titration period.

Safety assessments for all patients who received at least 1 dose of study medication (safety population) included continuous monitoring of adverse events, which were evaluated with regard to onset, severity, outcome, relationship to study medication, and action taken. Serious adverse events were monitored up to 30 days after the last dose of study medication and were followed up until the event resolved or became chronic or stable. Laboratory assessments, including evaluation of liver enzyme (alanine aminotransferase, aspartate aminotransferase, gamma-glutamyl transpeptidase, total alkaline phosphatase, and lactic dehydrogenase) levels, were performed at screening and at weeks 12 and 16.

### Statistical Analysis

All summaries were presented overall and also according to patients' previous phosphate binder use; phosphate binder naive, previous sevelamer HCL, calcium-based or 'other' binders. All analyses used the ITT population. Serum phosphorus, calcium, Ca × P product, PTH, daily dose, and tablet burden were summarized by visit within previous phosphate-binder therapy groups and overall. Changes from baseline were analyzed within previous phosphate-binder therapy groups and for the overall combined group using a paired *t *test. Results are presented as mean ± standard error of the mean (SEM).

Satisfaction and preference were summarized by visit within previous treatment groups and groups combined. Differences in satisfaction between baseline and week 12 and week 16 were analyzed using the McNemar test after combining responses of "strongly agree/agree" and "disagree/strongly disagree." Preference for lanthanum carbonate at weeks 12 and 16 was compared with preference for previous medication using a binomial proportion test. Treatment compliance was measured indirectly through questions provided on the physician and patient satisfaction questionnaires ("patient compliance," "rarely missed a dose," and "easy to take medication").

## Results

### Patients

This study enrolled 2763 patients at 223 sites. A detailed description of patient demographic characteristics is provided in Table 1. In the safety population (n = 2643), the most commonly reported reasons for study discontinuation were AEs (11.5%), withdrawal of consent (7.9%), and investigator decision (6.3%). All other reasons (alternate binder, death, and lost to follow-up) were each reported for < 3% of patients. Thirty-five (1.3%) patients did not have data on whether they completed the study or a recorded reason for withdrawal. The distribution of reasons for discontinuation in the ITT population was similar to that for the safety population.

**Table 1:** Patient Demographics

Patients enrolled, N	2763
Safety population, n	2643
ITT population, n	2520
Patients completed, n (%)	1751 (69.5)
Patients discontinued prematurely, n (%)	747 (29.6)
Sex, n (%)	
Men	1480 (58.7)
Women	1040 (41.3)
Age (years), mean (SD)	56.4 (14.3)
Race, n (%)	
White	1355 (53.8)
Black	1006 (39.9)
Other	158 (6.3)
Median time on dialysis, y (range)	2.2 (0-27)
Diabetic, n (%)	1244 (49.4)

ITT = intent to treat.

In the ITT population (n = 2520) at baseline, there were 105 (4.2%) patients naive to phosphate binder treatment, 1045 (41.5%) patients treated with calcium (all calcium-based products), 958 (38.0%) patients treated with sevelamer HCl, and 412 (16.3%) patients treated with 'other' (mostly combination) phosphate-binder therapies. Demographic characteristics were similar for each previous treatment group.

### Serum Phosphorus

**Figure 1:**
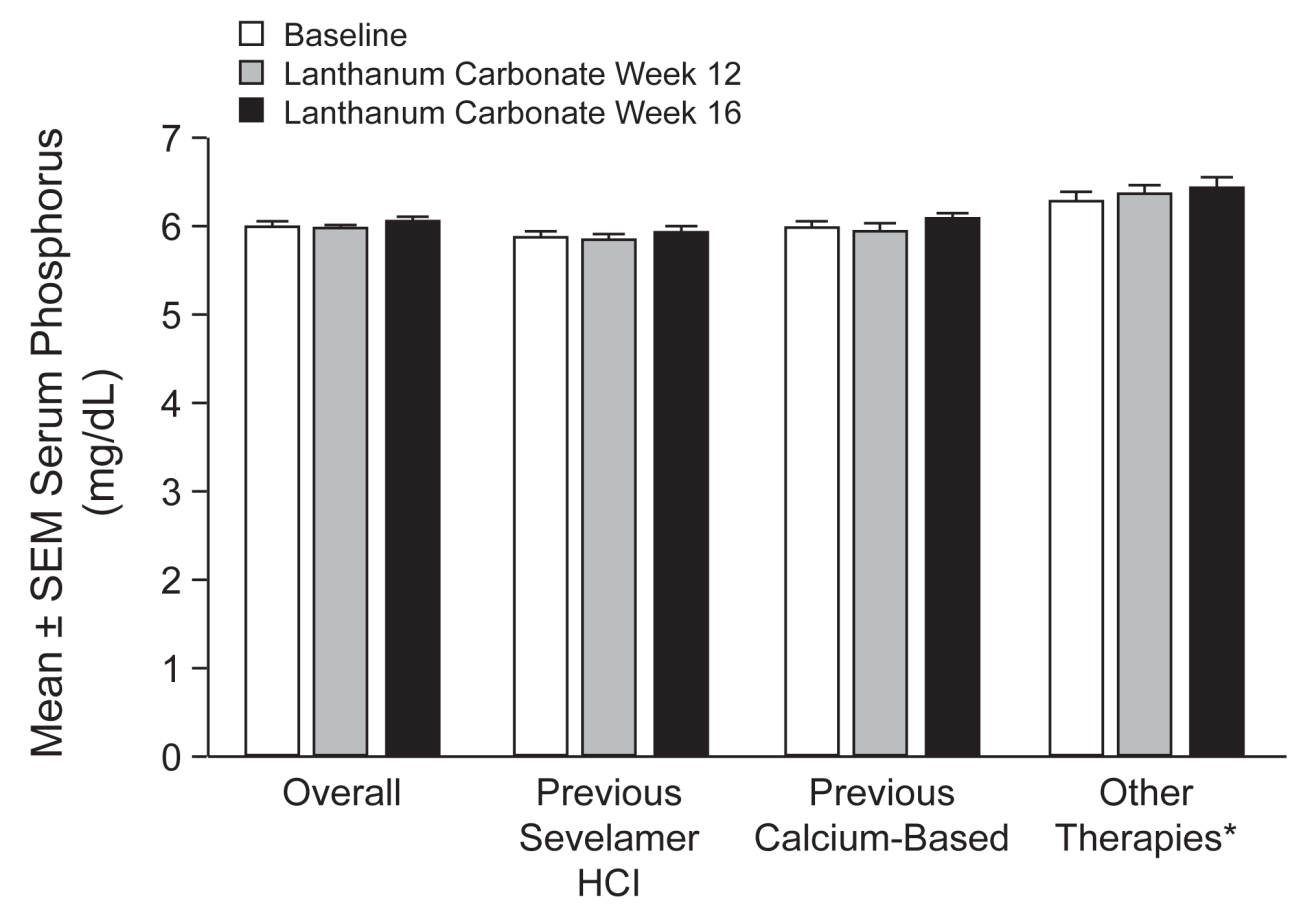
**Serum Phosphorus Levels**. Serum phosphorus levels at baseline and after converting to treatment with lanthanum carbonate (intent to treat population). SEM = standard error of the mean. *Mostly combination therapies; patients naive to treatment with phosphate binders (n = 105) are not shown.

**Figure 2:**
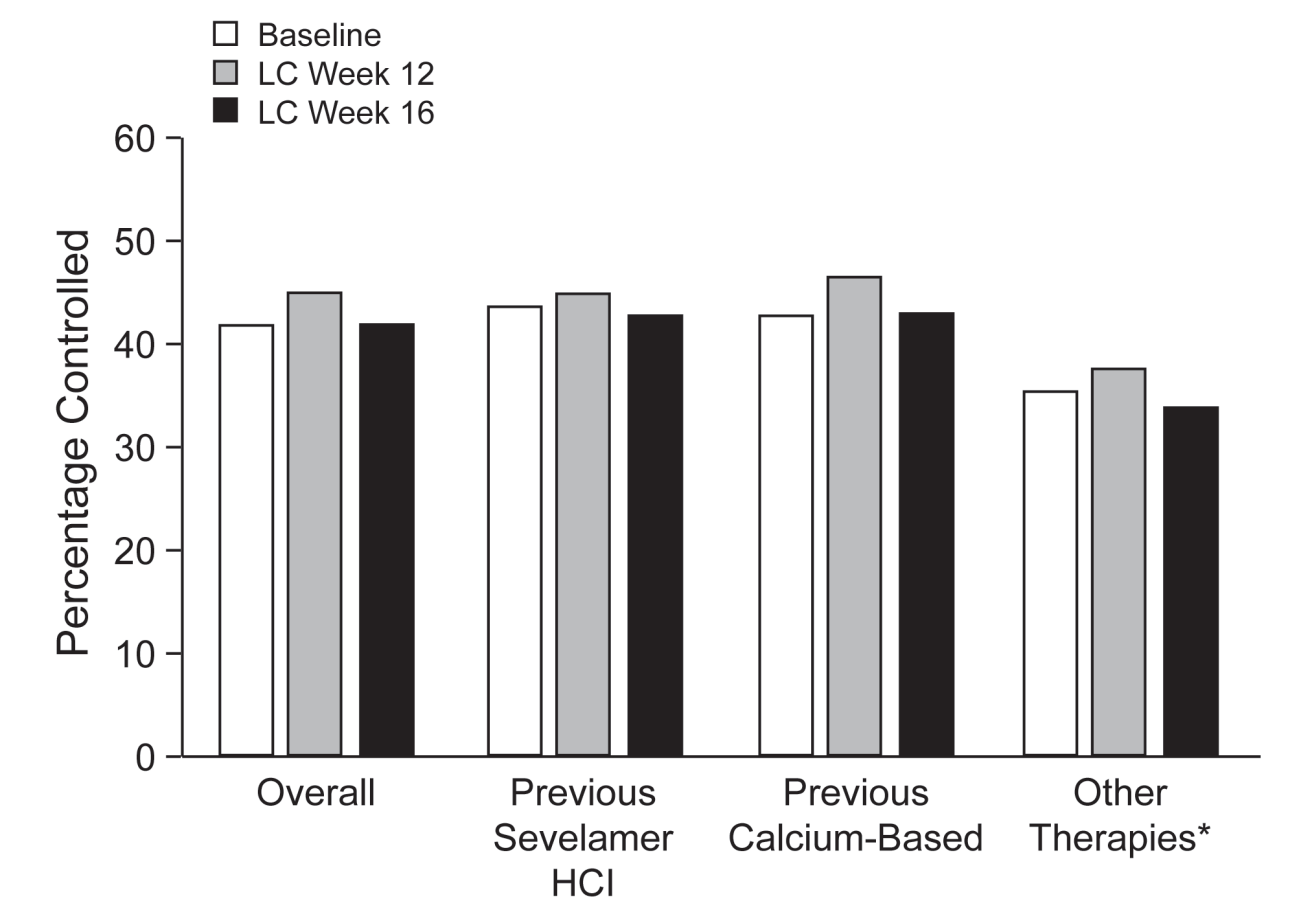
**Proportion of Patients With Controlled Serum Phosphorus Levels**. Percentage of patients with controlled serum phosphorus levels at baseline and weeks 12 and 16 of treatment with lanthanum carbonate (intent to treat population). Patients naive to treatment with phosphate binders (n = 105) are not shown. LC = lanthanum carbonate.

At baseline, mean serum phosphorus levels were 6.03 ± 0.04 mg/dL (ITT population). Overall, after converting to lanthanum carbonate monotherapy, patients' serum phosphorus levels throughout the 16-week study were similar to those achieved with treatment with their previous phosphate binder (Figure 1). The mean change from baseline was −0.06 ± 0.05 mg/dL at week 12 and 0.02 ± 0.05 mg/dL at week 16. Similar results were seen for the previous calcium, sevelamer, and 'other' treatment groups. For the binder-naïve group, statistically significant reductions were seen at weeks 12 and 16 (−0.41 ± 0.19 mg/dL and −0.62 ± 0.19 mg/dL at weeks 12 and 16 respectively). Similar proportions of patients achieved serum phosphorus control (≤5.5 mg/dL) at baseline (41.8%) and maintained control during treatment with lanthanum carbonate overall and in each treatment group (overall group: week 12, 44.9%; week 16, 41.6%). In the binder-naïve group the percentage with phosphorous control increased from 36.5% at baseline to 58.7% at week 12 and 50.6% at week 16. (Figure 2).

### Satisfaction and Preference

**Figure 3:**
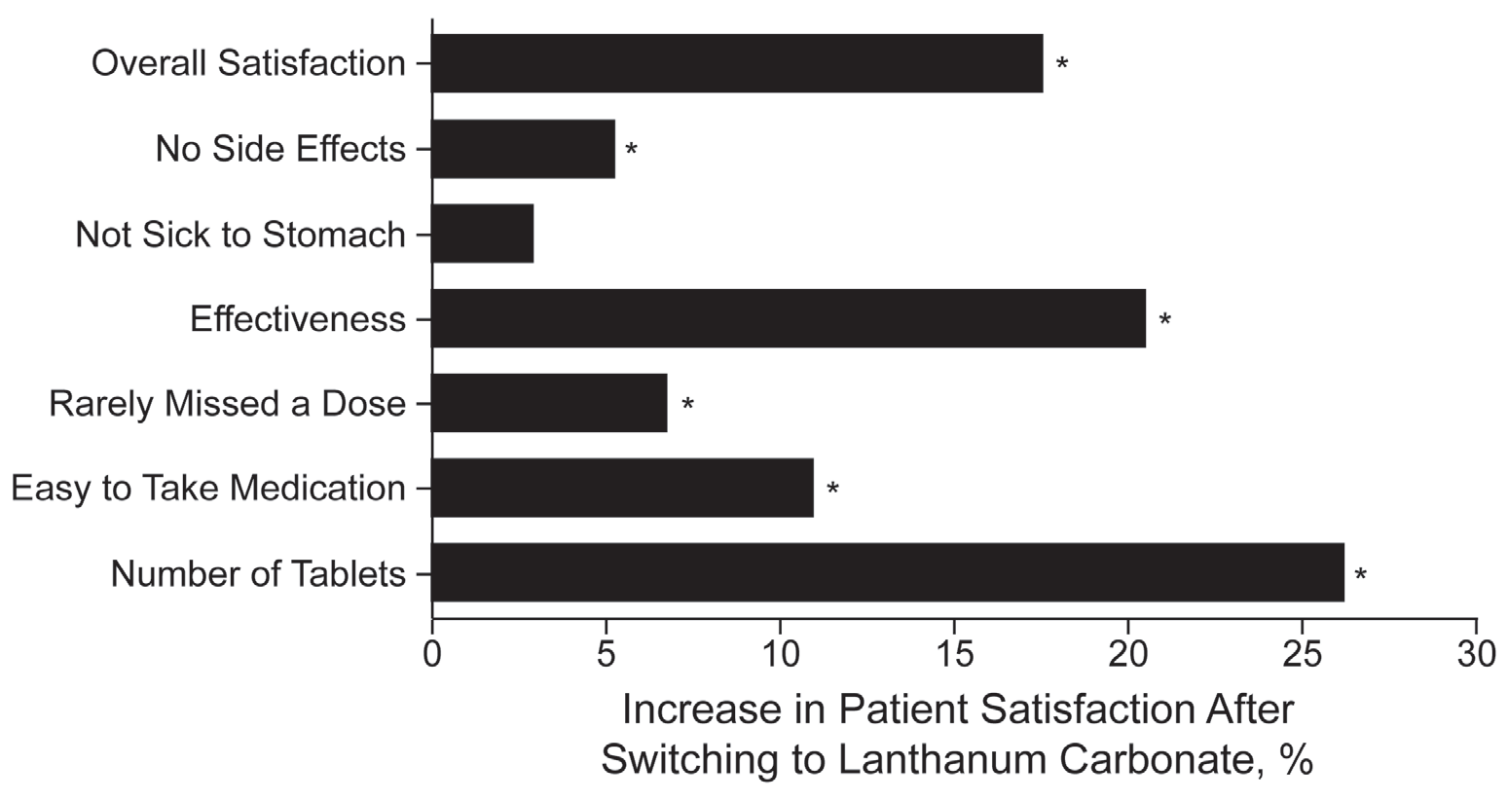
**Patient Satisfaction**. Change in patient satisfaction after 12 weeks of treatment with lanthanum carbonate (intent to treat population). Patients responded positively with "agree" or "strongly agree." *P< 0.0001 from chi-square test on change from baseline.

**Figure 4:**
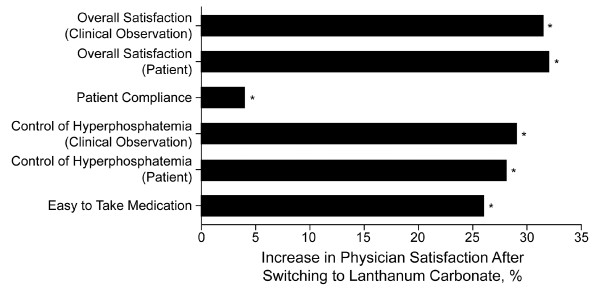
**Physician Satisfaction**. Change in physician satisfaction after 12 weeks of treatment with lanthanum carbonate (intent to treat population). Physicians responded positively with "agree" or "strongly agree." *P< 0.0001 from chi-square test on change from baseline for all parameters.

Assessments of patient and physician satisfaction revealed that satisfaction at 12 weeks and 16 weeks of treatment with lanthanum carbonate was markedly improved compared with baseline values when patients were using their previous phosphate binder (Figures 3 and 4). Patient satisfaction was statistically significantly greater after treatment with lanthanum for each of the separate domains for which satisfaction was measured, except for stomach sickness and experiencing other side effects (significant at week 12 but not week 16). The increase from baseline in overall satisfaction was greater for physicians than for patients; similar increases in satisfaction were observed at week 16 (data not shown). Analysis of changes in satisfaction for patients who were treated previously with sevelamer HCl or calcium-based phosphate binders revealed similar overall percentage changes. Overall physician satisfaction increased by 34% and 29% (based on clinical observation) for patients who were treated previously with calcium-based binders and sevelamer HCl, respectively, after 12 weeks of treatment with lanthanum carbonate. Overall patient satisfaction increased by 14% and 18% for patients who were treated previously with calcium-based binders and sevelamer HCl, respectively.

**Figure 5:**
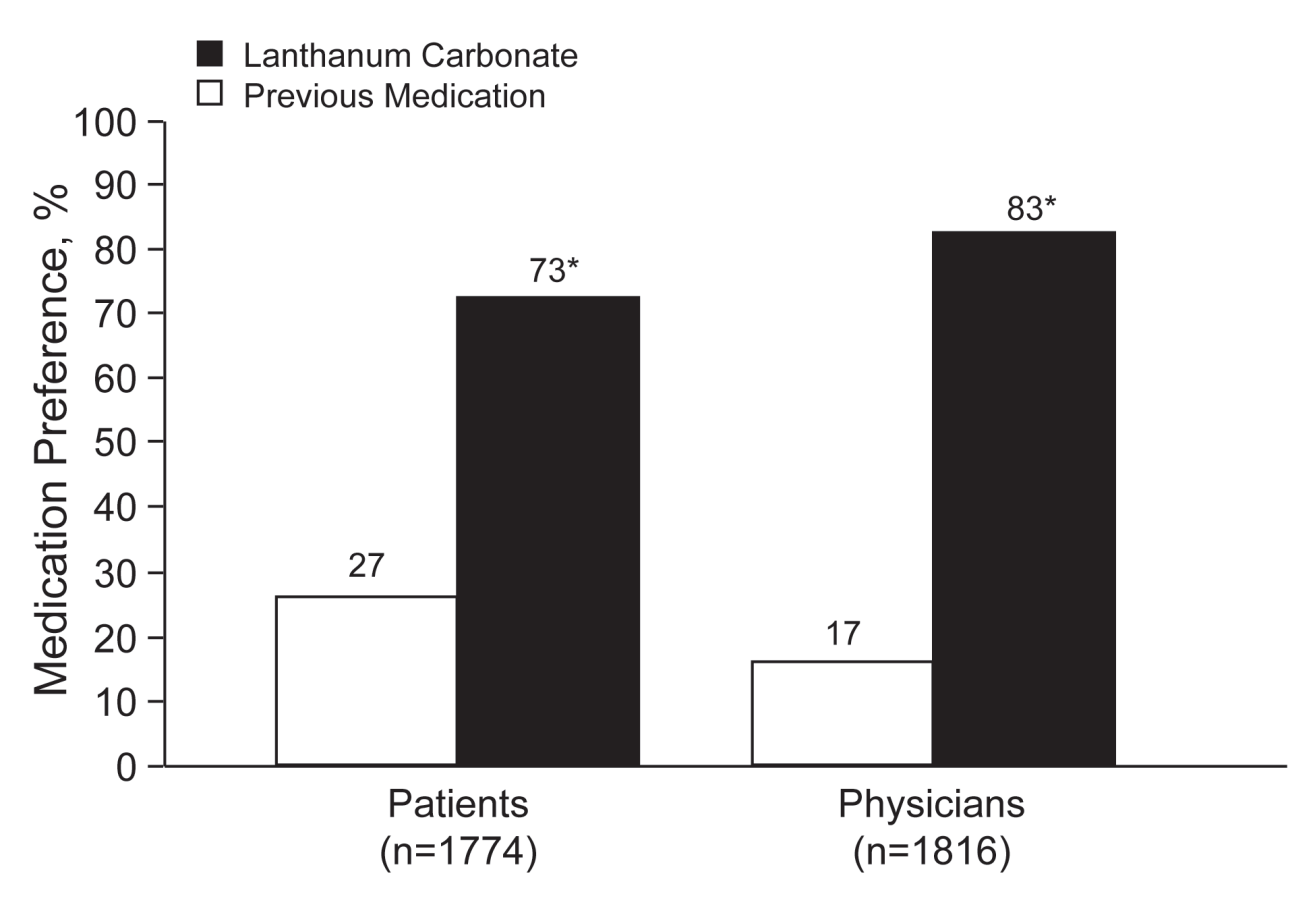
**Overall Medication Preference**. Patient and physician overall medication preference at week 12 of treatment with lanthanum carbonate (intent to treat population). *P< 0.0001 from binomial test procedure with null hypothesis; proportion = 0.5.

Seventy-three percent of patients and 83% of physicians participating in the study preferred lanthanum carbonate over previous phosphate-binder medication at week 12 (Figure 5), with a similar trend at week 16. Statistically significant differences in preference compared with previous therapy were observed at weeks 12 and 16 for all domains encompassed within the preference questionnaire, including the number of tablets, ease of taking medication, symptom control, and adverse effects for patients and available dosage forms, effectiveness, adverse effects, and patient adherence for physicians.

### Daily Dose and Tablet Burden

**Figure 6:**
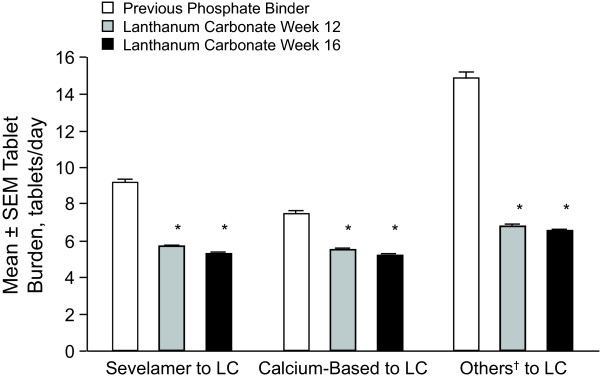
**Tablet Burden Comparison**. Comparison of tablet burden of previous treatment with phosphate binders and treatment with lanthanum carbonate (intent to treat population). LC = lanthanum carbonate; SEM = standard error of the mean. *P< 0.0001 for week 12 and 16 values from paired *t *test on change from baseline values.^†^The "Others" category consists mainly of combination therapies.

There were significant reductions for all previous treatment groups in tablet burden (*P*< 0.001; Figure 6) and daily dose (*P*< 0.0001) at weeks 12 and 16 of treatment with lanthanum carbonate. The mean daily dose was significantly reduced from 7.2 g at baseline to 2.8 and 2.7 g at weeks 12 and 16, respectively (*P*< 0.0001). Reductions in daily tablet burden at week 12 ranged from 2.2 ± 0.15 pills for patients previously receiving calcium-based therapy (week 12) to 8.4 ± 0.40 pills for patients previously receiving 'other' treatments. Similar reductions were seen at week 16.

For patients previously treated with calcium acetate, sevelamer HCl, or 'other' there were significant (*P*< 0.0001) reductions in the dose of phosphate binder required to maintain serum phosphorus control when patients were converted to treatment with lanthanum carbonate. The mean reduction in total daily dose ranged from 2.6 g/d to 8.1 g/d. At week 12, the 'others' group had the greatest mean reduction of 8.1 g/d while the calcium group had the lowest reduction of 2.6 g/d. Similar reductions were seen at week 16. The mean daily doses of lanthanum carbonate required to maintain serum phosphorus levels were similar across all 3 groups previously treated with phosphate binders, despite the wide variation in previous doses. For example, the mean ± SEM dose of calcium previously used was 5.4 ± 0.09 g/d and the mean ± SEM dose of lanthanum carbonate at week 12 was 2.7 ± 0.04 g/d. Those patients previously treated with sevelamer received 2.8 ± 0.04 g/d of lanthanum carbonate at week 12 compared with 7.6 ± 0.12 g/d of sevelamer at baseline. Serum phosphorus levels were stable from baseline through week 16 for all groups despite the markedly lower doses of lanthanum carbonate relative to previous medication.

### Laboratory Assessments

The changes in corrected Ca, Ca × P, and PTH from baseline are presented in (Table 2). Overall there were statistically significant decreases in the serum calcium levels and the changes in Ca × P product were consistent with this. There was a statistically significant increase in PTH after a change to treatment with lanthanum carbonate.

**Table 2:** Laboratory Values at Baseline and change from Baseline at Weeks 12 and 16 of Lanthanum Carbonate Treatment (ITT Population)

Assessment	**Corrected Serum Ca**, mg/dL	**Ca × P Product**, mg^2^/dL^2^	**Biointact PTH**, pg/mL
Baseline	9.49 ± 0.06	56.16 ± 0.49	241.88 ± 7.15
LC change from baseline to week 12	−0.13 ± 0.06*	−1.15 ± 0.62	59.19 ± 7.33*
LC change from baseline week 16	−0.09 ± 0.06	−0.30 ± 0.66	49.30 ± 7.70*

Ca = calcium; Ca × P = calcium × phosphorus; ITT = intent to treat; LC = lanthanum carbonate; PTH = parathyroid hormone.

All data are mean ± standard error of the mean. *P≤ 0.05.

### Safety and Tolerability of Converting to Lanthanum Carbonate

The incidence of adverse events reported during the study was consistent with the known safety profile for lanthanum carbonate. Among patients in the safety population, adverse events were reported by 952/2643 (36.0%) patients; 327 (12.4%) patients had adverse events which led to discontinued study participation. Most adverse events were mild or moderate, with severe events reported by 12.1% of all patients. The most common adverse events included nausea (7.9%), diarrhea (5.4%), and vomiting (5.0%). Of all adverse events, 12.1% were considered probably related to treatment and 5.4% were deemed possibly related to treatment. Serious adverse events were reported by 14.9% of patients, most were considered by the investigator to be unrelated to study medication.

There were no statistically significant changes from baseline in liver enzymes (except alkaline phosphatase). For alkaline phosphatase, mean ± SD levels at screening were 111.4 ± 77.9 U/L; increases from screening of 5.3 ± 70.6 and 6.8 ± 74.8 U/L were observed at weeks 12 and 16, respectively. However, the clinical significance of this result could not be determined because of the large standard deviations.

## Discussion

Hyperphosphatemia is a serious consequence of advanced kidney failure. A majority of patients with ESRD/stage 5 chronic kidney disease require a phosphate binder as part of their treatment regimen, and in some treatment may be required as early as stage 3. In the study described here, patients with ESRD were converted from previous treatments to lanthanum carbonate monotherapy in a clinical practice setting. Patients received prescriptions for study medication from their nephrologist and obtained lanthanum carbonate tablets from local pharmacies. Compared with most other clinical trials in which patients receive study medication at the trial site, this study design more accurately represents routine clinical activities, thereby increasing the applicability of the results to standard practice. The study, however, did not include active comparator or placebo arms, either of which may have strengthened the results.

Patients were converted to lanthanum carbonate monotherapy without a washout period from previous phosphate-binder regimens, which consisted of calcium-based phosphate binders (41.5%), sevelamer (38.0%), 'other' (combinations of different binders; 16.3%), and binder naïve (4.2%), respectively. The conversion to lanthanum carbonate (250-mg and 500-mg tablets) resulted in maintenance of serum phosphorus levels. The mean change from baseline was −0.06 ± 0.05 mg/dL at week 12 and 0.02 ± 0.05 mg/dL at week 16. At baseline, 41.8% of patients achieved the KDOQI-recommended level of control (≤5.5 mg/dL); 44.9% and 41.6% achieved control at treatment weeks 12 and 16, respectively. Slightly fewer than half of patients achieved serum phosphorus control, reflecting disease severity. This illustrates the difficulty of attaining treatment targets in patients with ESRD, who frequently have comorbid conditions such as diabetes and hypertension.

Patients and physicians reported increases in their levels of satisfaction after converting to treatment with lanthanum carbonate. Physician satisfaction with observed efficacy (control of hyperphosphatemia, clinical observation) increased with lanthanum carbonate treatment at week 12, even though control of serum phosphorus levels did not improve significantly over baseline levels. Patients and physicians indicated a statistically significant preference for lanthanum carbonate over previous phosphate-binder medications for the majority of domains.

Tablet burden has been reported to influence patient adherence with phosphate-binder therapy [8,9]. In a study conducted at 3 dialysis units across the United States, higher pill burden was associated with lower health-related quality of life and was not found to improve control of serum phosphate levels [14]. In the current study, a careful evaluation of tablet burden and daily dose was performed. Significant reductions were observed for the total daily dose and tablet burden; mean dose reductions ranged from 2.6 to 8.1 g/d required to control serum phosphorus and the mean reduction in overall daily tablet burden ranged from 2.2 to 8.4 pills per day at week 12, with similar results at week 16. Although treatment with lanthanum carbonate resulted in reduced tablet burden, comparable levels of serum phosphorus control were maintained throughout the study. This observation plus findings from Chiu et al [14] suggest that although tablet burden may improve treatment compliance and quality of life, lowering or raising the number of pills does not necessarily influence phosphate levels. Depending on the previous phosphate-binder regimen, the patients in this study were taking, on average, between 8 and 15 pills per day at baseline, compared with an average of approximately 6 tablets daily of the original larger lanthanum carbonate formulation (no longer manufactured) to control serum phosphorus at study week 16. Although patients had a wide dose range of previous medication at baseline, the average daily dose of lanthanum carbonate required to control serum phosphorus was between 2.5 and 3.0 g. This dose of lanthanum carbonate (~3.0 g/d) will allow for dosing of 1 tablet per meal with 1000 mg tablets [15]. The large difference between the doses of lanthanum carbonate and previous binders may be explained, in part, by in vitro data that show that lanthanum carbonate binds phosphate ions with a higher affinity compared with other phosphate binders [13]. In addition, unlike sevelamer and calcium-based phosphate binders, lanthanum carbonate binds phosphate within the entire pH range found in the gastrointestinal tract [13].

Although this study demonstrates that lanthanum carbonate is effective at controlling serum phosphorus with a reduced tablet burden in patients with ESRD, several noteworthy limitations exist. The results from the study would have been strengthened if an active comparator arm or a placebo arm had been included. However, the present study was designed to assess whether results obtained from clinical trials of lanthanum carbonate could be replicated in the clinical practice setting. The use of the TrialCard system, in which patients receive free medication, may have biased patient opinion when comparing with previous medications that likely required copayment. Physicians were compensated for their time and involvement in this study, and thus, physician bias with regard to product preference and satisfaction cannot be excluded. Lastly, patient compliance with the treatment regimen was not stringently monitored. However, indirect measures of compliance, such as improved patient satisfaction associated with easy to use study medication (Figure 3) and rarely missing a dose (Figure 3), as well as improved physician satisfaction with compliance (Figure 4), suggest that patient compliance was not a significant issue.

Although there was no comparator arm in this study, the efficacy and tolerability of lanthanum carbonate have been well documented in previous studies [11,12,16-18]. Changes in Ca × P product were consistent with statistically significant decreases in the serum calcium levels. There was a statistically significant increase in PTH after a change to treatment with lanthanum carbonate, thus it is reasonable to suggest that the increase in alkaline phosphatase observed at weeks 12 and 16 may have been due to normalization of bone formation rate (ie, increased bone alkaline phosphatase fraction).

## Conclusions

Lanthanum carbonate monotherapy offers effective serum phosphorus control comparable to that of other phosphate binders, with a reduced daily dose and tablet burden for the patient compared with other phosphate binders, including sevelamer HCl and calcium salts, as monotherapy or in combination. Conversion to treatment with lanthanum carbonate from other phosphate binders resulted in improved patient and physician satisfaction. The majority of patients and physicians preferred treatment with lanthanum carbonate over previous treatment with phosphate binders. Lanthanum carbonate may offer advantages in the treatment of hyperphosphatemia in ESRD by reducing tablet burden, which translates into a simplified dosing regimen that may have a positive impact on patient adherence and rates of serum phosphorus control in the clinic. Additionally, this reduction in tablet burden with lanthanum carbonate may positively impact adherence with other nonbinder medications prescribed for ESRD patients, who often are managing other comorbid conditions.

## Competing interests

Dr. Matalon declares no competing interests. Dr. Michelis has received compensation from Shire Pharmaceuticals for performing clinical research. Dr. Vemuri has worked as a consultant for Shire Pharmaceuticals during clinical research trials.

## Authors' contributions

AM and MM participated in acquisition of the data. MM and NV participated in the analysis and interpretation of the data. All authors helped to draft the manuscript and have read and approved the final manuscript.

## Pre-publication history

The pre-publication history for this paper can be accessed here:

http://www.biomedcentral.com/1471-2369/12/49/prepub
